# Use of Dialogue to Uncover Perceptions of Preconception Care From Women of Reproductive Age in the Sampled Districts of Limpopo Province

**DOI:** 10.1155/jp/4942818

**Published:** 2026-07-03

**Authors:** N. P. Ndou, T. Malwela, M. S. Maputle, N. S. Raliphaswa, A. Samie, L. Mabasa

**Affiliations:** ^1^ Department of Advanced Nursing Sciences, Faculty of Health Sciences, University of Venda, Thohoyandou, South Africa, univen.ac.za; ^2^ Department of Biochemistry and Microbiology, Faculty of Sciences, Engineering and Agriculture, University of Venda, Thohoyandou, South Africa, univen.ac.za; ^3^ Biomedical Research and Innovation Platform (BRIP), South Africa Medical Research Council, Cape Town, South Africa

**Keywords:** perceptions, perinatal outcomes, preconception care, women of reproductive age

## Abstract

**Background:**

Preconception care (PCC) includes biomedical, behavioral, and social health interventions provided before conception to improve maternal and neonatal health outcomes. Despite its importance, the utilization of PCC remains limited in many settings, including parts of South Africa. Understanding women′s perceptions of PCC is essential for improving service uptake and implementation. This study explored and described the perceptions of women of reproductive age regarding the uptake and utilization of PCC services in selected districts of Limpopo province, South Africa.

**Methods:**

A qualitative, exploratory, and descriptive design was used. The study was conducted in primary healthcare clinics and community health centers in three districts of Limpopo province: Capricorn, Mopani, and Vhembe. Purposive sampling was used to recruit 51 women aged 19–35 years. Data were collected through eight focus group discussions (FGDs) consisting of six to eight participants per group. Discussions were conducted in Tshivenda, Xitsonga, and Sepedi, audio‐recorded with consent, and transcribed verbatim. Data were analyzed using Tesch′s eight‐step open coding method. Trustworthiness was ensured through member checking, peer debriefing, and the maintenance of an audit trail. Ethical guidelines were adhered to.

**Results:**

Two main themes emerged. First, participants perceived PCC as a valuable service that can promote healthy pregnancies and improve maternal health outcomes. Participants emphasized the need for early education and access to PCC services. Second, several barriers to PCC utilization were identified, including limited awareness of PCC, fear of HIV testing, cultural norms surrounding pregnancy disclosure, and lack of partner support.

**Conclusion:**

Although women recognized the benefits of PCC, several sociocultural and structural barriers limited its utilization. Increasing awareness, promoting partner involvement, strengthening health system integration, and improving culturally sensitive health education may enhance PCC uptake in Limpopo province.

**Notes**

To ensure a healthy pregnancy and the best possible outcomes for the mother and the unborn child, preconception care (PCC) focused on enhancing women′s health and well‐being before becoming pregnant may be the best option.

## 1. Introduction

PCC refers to biomedical, behavioral, and social health interventions provided before conception to improve maternal, newborn, and child health outcomes. In 2013, the World Health Organization (WHO) issued comprehensive guidelines emphasizing the importance of addressing women′s physical, mental, and social health before pregnancy to ensure optimal pregnancy outcomes [1]. PCC is aimed at identifying and modifying risk factors such as chronic diseases, infections, nutritional deficiencies, substance use, and psychosocial stressors before conception occurs. By intervening during this critical window, PCC strengthens the continuum of care and reduces preventable adverse maternal and neonatal outcomes. Research on PCC identifies gaps in its implementation and education to reduce pregnancy risks [2, 3].

Failure to implement PCC effectively may result in missed opportunities to detect and manage health risks before pregnancy. Conditions such as diabetes mellitus, hypertension, infections, poor nutrition, alcohol use, tobacco exposure, and psychosocial stress are associated with adverse perinatal outcomes, including miscarriage, low birth weight, congenital abnormalities, and maternal complications [4–6]. Therefore, comprehensive counseling and risk screening during the preconception period are essential to improving both maternal and neonatal health indicators. Globally, maternal mortality remains unacceptably high, estimated at 223 deaths per 100,000 live births, making the Sustainable Development Goal (SDG) target of reducing maternal mortality to less than 70 per 100,000 live births by 2030 difficult to achieve [6]. In South Africa, maternal mortality declined from 105.9 per 100,000 live births in 2019 to 88.0 per 100,000 in 2020, reflecting progress toward this goal. However, provincial disparities persist. Limpopo province reported a maternal mortality ratio of 97.8 per 100,000 live births in 2020, alongside a perinatal mortality rate of 28 per 1,000 births [7]. These statistics highlight the continued need for preventive strategies, including PCC, to address modifiable pre‐pregnancy risk factors. Studies show how complex the factors affecting perinatal outcomes are, including genetics, lifestyle choices, infections, chronic diseases pre‐existing before pregnancy, extremely young or advanced age, and malnutrition in the mother [8]. Although strategies such as basic antenatal care and family planning are in place to reduce poor perinatal outcomes [9, 10], these interventions typically begin after conception. PCC fills this gap by targeting risk factors before pregnancy occurs. Despite its recognized importance, studies globally and locally suggest that implementation and utilization of PCC remain suboptimal. Research in the Netherlands and Nigeria indicates generally positive perceptions and acceptability of PCC among women [11, 12]. In South Africa, van der Zee et al. [13] reported favorable perceptions of PCC, yet Ojifinni and Ibisomi [14] demonstrated that positive attitudes do not necessarily translate into utilization. This discrepancy suggests that contextual and social factors influence uptake beyond mere awareness or approval.

In Limpopo province, previous research has identified poor implementation of PCC services [15]. However, these studies did not explore in depth how women of reproductive age perceive PCC or how sociocultural, relational, and systemic factors shape their decisions to utilize such services. Without understanding women′s perceptions, beliefs, fears, and lived realities, efforts to improve PCC coverage may remain ineffective [16]. Additionally, factors such as HIV‐related stigma, cultural norms surrounding pregnancy disclosure, and partner support may uniquely influence service uptake in this context. Thus, a critical knowledge gap exists regarding women of reproductive age′s perceptions of PCC utilization in Limpopo province [17]. However, in addition to traditional facility‐based services, contemporary digital tools are increasingly recognized as important platforms for delivering PCC information. Numerous studies demonstrate that women of reproductive age frequently rely on the internet as their primary source of health information, including reproductive and pregnancy‐related guidance [18].

Digital platforms that provide validated, up‐to‐date, and evidence‐based content, therefore, represent a strategic opportunity to improve access to PCC and enhance informed decision‐making. Montanaro et al. [18] proposed a technology‐based model for PCC delivery integrated within primary care and supported by public health systems, demonstrating the feasibility of digital approaches to expand service reach. Similarly, Hristova‐Atanasova et al. [19] reported that women′s attitudes toward preconception health are influenced by access to reliable health information, underscoring the importance of structured and credible information channels. More recently, Nurleli et al. [20] highlighted the effectiveness of digital health interventions in improving pregnancy quality among preconception women, emphasizing the potential of technology‐based models to strengthen reproductive health outcomes. Despite growing evidence supporting digital and technology‐assisted PCC delivery, little is known about whether women in Limpopo province perceive digital platforms as accessible or trustworthy sources of preconception information.

Understanding women′s perceptions of PCC within both traditional healthcare and emerging digital contexts is therefore essential for designing comprehensive, contextually appropriate strategies to improve uptake and utilization [18]. It was against this background that the current study was conducted in selected districts of Limpopo province to explore and describe the perceptions of women of reproductive age regarding the uptake and utilization of PCC. The findings are aimed at informing strategies to improve public awareness, strengthen community engagement, and ensure effective integration of PCC into primary healthcare services. The purpose of this study was to explore and describe the perceptions of women of reproductive age regarding the uptake and utilization of PCC services in selected districts of Limpopo province.

## 2. Material and Methods

This study adopted a qualitative, exploratory, and descriptive research design. A qualitative methodology was considered appropriate because the study sought to explore and understand the perceptions, beliefs, and lived experiences of women of reproductive age regarding the uptake and utilization of PCC. Since perceptions are socially constructed and influenced by contextual, cultural, and relational factors, they cannot be adequately captured through quantitative measurement alone [21].

An exploratory design was selected because limited research has been conducted in Limpopo province on women′s perceptions of PCC. The descriptive component enabled a detailed account of participants′ perceptions in their own words, thereby providing context‐rich insights into the phenomenon under investigation. This design was therefore directly aligned with the research question [21]: *What are the perceptions of women of reproductive age regarding the uptake and utilization of preconception care services in selected districts of Limpopo province?*

### 2.1. Study Setting

The study was conducted in primary healthcare clinics and community health centers (CHCs) located in three districts of Limpopo province: Capricorn, Mopani, and Vhembe. These districts were selected to ensure representation across the province′s different geographic areas. The facilities provide reproductive health, maternal health, and general primary healthcare services to women of reproductive age [22].

### 2.2. Study Population and Sampling

The study population consisted of women of reproductive age (19–35 years) who attended clinics or CHCs for reproductive health services, maternity care, or other healthcare services, regardless of previous pregnancy history. A nonprobability purposive sampling technique was employed [22]. Purposive sampling was appropriate because participants were intentionally selected based on their relevance to the research question—namely, being women of reproductive age within the selected districts. Homogeneous sampling ensured that participants shared key characteristics (age range, reproductive potential, and geographic location), facilitating focused exploration of shared perceptions regarding PCC. A total of 51 women participated in the study. Inclusion criteria were women aged 19–35 years, residing in Capricorn, Mopani, or Vhembe districts, attending selected clinics or CHCs during the data collection period, and willing to provide informed consent. Exclusion criteria included women outside the specified age range, women not residing in the selected districts, and those unwilling to participate [21–23].

### 2.3. Data Collection

Data were collected through eight focus group discussions (FGDs) comprising six to eight participants per group. FGDs were chosen because they facilitate interaction, collective reflection, and shared meaning‐making among participants. This method was particularly appropriate for exploring perceptions influenced by social norms, cultural beliefs, and relational dynamics. The FGDs were conducted in quiet rooms within the health facilities to ensure privacy and minimal disruption. The researcher and a trained moderator facilitated the discussions. The moderator ensured that discussions remained focused while allowing participants to freely express their views. The central guiding question was [22]: “What are your perceptions regarding the uptake and utilization of preconception care services?”

Follow‐up probes were used to deepen responses and clarify emerging issues. Discussions were conducted in participants′ native languages (Tshivenda, Xitsonga, and Sepedi) to enhance comfort, clarity, and depth of expression. With participants′ consent, discussions were audio‐recorded. Field notes were also taken to document nonverbal cues, group dynamics, and contextual observations. Data saturation was reached after six FGDs; however, two additional FGDs were conducted to confirm saturation and enhance credibility [21].

### 2.4. Data Management and Analysis

All FGDs were audio‐recorded with participants′ consent and transcribed verbatim in the original languages of data collection (Tshivenda, Xitsonga, and Sepedi) by a trained transcriptionist fluent in those languages. Data were analyzed using Tesch′s eight‐step open coding method [21], which allows themes to emerge inductively from participants′ narratives. The analysis included the following steps: a systematic qualitative procedure. First, the researchers familiarized themselves with the data by repeatedly reading all transcripts and noting initial impressions. Next, inductive coding was conducted by examining transcripts line by line and assigning descriptive codes to meaningful segments of text. A preliminary code list was then developed by grouping similar codes and defining them to ensure consistent use.

To improve reliability, an independent co‐coder analyzed selected transcripts using the preliminary code list. A consensus meeting was held to compare coding decisions, discuss disagreements, and refine codes until agreement was reached. Afterward, related codes were grouped into broader categories, which were further synthesized into themes and subthemes representing key patterns in participants′ responses [23]. The themes were supported with representative quotations. Finally, the themes were verified and finalized by reviewing them across all transcripts. Member checking with selected participants and maintaining an audit trail helped ensure credibility, consistency, and transparency in the analysis process. Verbatim transcription included pauses, emotional expressions, and significant nonverbal cues (e.g., laughter and prolonged silence), which were indicated in brackets to preserve contextual meaning. Following transcription, the original‐language transcripts were translated into English by a professional bilingual linguist with expertise in the respective languages and familiarity with health‐related terminology [23].

### 2.5. Measures to Ensure Trustworthiness

Trustworthiness was ensured through the criteria of credibility, transferability, dependability, and confirmability [24]. Credibility was enhanced through prolonged engagement, data saturation, member checking, and audio recordings. Member checking was conducted in two stages to ensure accuracy of the findings. First, at the end of each FGDs, the moderator summarized key points and participants confirmed or clarified the interpretations [24]. Second, preliminary themes were presented to a subset of participants from each district in a follow‐up session. Participants confirmed that the themes reflected their views, and minor clarifications were incorporated into the final analysis. Peer debriefing involved two independent qualitative research experts who reviewed the coding framework, theme development, and selected transcript excerpts. They questioned assumptions, offered alternative interpretations, and discussed findings with the researcher until consensus was reached. This process helped reduce researcher bias and strengthened the rigor of the analysis [24].

### 2.6. Ethical Considerations

Ethical clearance (Ref: FHS/22/PDC/05/2504) was obtained from the University of Venda Research Ethics Committee. Permission to conduct the study was granted by the Department of Health Ethics Committee and the district management authorities. Participation was voluntary. Informed consent was obtained and signed by all participants. Participants were informed of their right to withdraw at any stage without penalty. Anonymity and confidentiality were maintained by using codes instead of names or facility identifiers [21]. The recordings were stored in the cloud with the encryption code, and access is given to the promoters only [22].

## 3. Results

### 3.1. Demographic Data of Women of Childbearing Age (WCA)

The demographic profile (Table 1) shows that most participants (25 out of 51) were aged 26–30 years, and a substantial proportion had already had two children. This suggests that the study primarily captured the perspectives of women in midreproductive age with prior pregnancy experience. Their insights are likely shaped by both personal reproductive history and exposure to maternal health services, which may influence their understanding and perception of PCC. The relatively even distribution across the three districts (Capricorn, Mopani, and Vhembe) enhances the representativeness of the findings within Limpopo province and allows for insights that may reflect shared regional experiences rather than location‐specific anomalies. The age and parity distribution also highlight potential variation in PCC needs and awareness: Younger women or those without children may require more foundational information, whereas women with prior pregnancies may have knowledge shaped by previous antenatal encounters. This demographic context informs the interpretation of themes, particularly those related to awareness, perceived benefits, and utilization challenges.

**Table 1:** Demographic data of women of childbearing age (WCA).

Number of FGDs	Age	Parity	Districts
8	19–25 = 17	0 = 3	Capricorn = 18
	26–30 = 25	1 = 8	Mopani = 17
	30–35 = 9	2 = 25	Vhembe = 16
		3 = 15	
Total	51	51	51

### 3.2. Themes and Subthemes Derived From FGDs

Table 2 outlines the themes and subthemes that emerged from the FGD data. These themes reflect participants′ perceptions and experiences regarding using PCC in their communities, particularly for WCA.

**Table 2:** Themes and subthemes derived from focus group discussions (FGDs).

No.	Themes	Subthemes
1.	PCC is perceived as a valuable service	1.1 PCC was seen as being beneficial to women of childbearing age, thus a need for it to be available
		1.2 Women to be informed early about the benefits of PCC

2.	Identification of potential challenges regarding PCC utilization	2.1 Lack of awareness about PCC
		2.2 Fear of knowing about their HIV status
		2.3 Cultural issues regarding pregnancy may have an impact on PCC utilization
		2.4 Lack of support from spouse

#### 3.2.1. Theme 1: PCC Was Viewed as a Valuable Service

PCC was viewed as important by WCA. Two subthemes emerged under PCC that were viewed as valuable services; namely, PCC was seen as beneficial to WCA, thus necessitating its availability and informing women of its benefits.

##### 3.2.1.1. Subtheme 1.1: PCC Is Beneficial and Should Be Accessible

Participants consistently emphasized the perceived health benefits of PCC, linking access to preventive measures, early health assessment, and preparation for healthy pregnancies. For example, several participants stated:

“…FGD 8: Participants 1 and 6 and FGD 2: Participants 1, 2, and 5 said,” “This [PCC] is important as it can help women know their health status and lead a healthy lifestyle as the nurses will advise them.”

“…FGD 4: Participants 1 and 5, FGD 3: Participants 3 and 5, and FGD 7: Participants 1 and 6 shared similar ideas as denoted by this quote, “…The PCC will help women to be healthy before they fall pregnant, thus likely to be healthy during pregnancy as well…”

“…FGD 1: Participants 3 and 6 and FGD 5: Participants 1 and 4 again said,” “…The PCC is important, and every woman should be able to access the service in the clinic…”

This statement reflects that participants view PCC not only as a medical check but also as an educational and preventive tool. Their responses suggest an understanding of PCC as a mechanism for empowerment and risk reduction, particularly for managing potential health conditions before pregnancy. Across groups, participants echoed the need for PCC availability in clinics, reinforcing the interpretation that access is seen as a prerequisite for uptake. By synthesizing these responses, it becomes clear that the perceived value of PCC is both instrumental (preventing complications) and informational (guiding health behaviors).

##### 3.2.1.2. Subtheme 1.2: Early Information Is Critical

Participants emphasized the importance of informing women about PCC early, even before pregnancy intentions are realized. One participant noted that:

“…FGD 3: Participants 1 and 4 and FGD 6: Participants 1 and 3 had this to say,” “…umm, this is important, and I think young women should be informed about this PCC while still young before they even start having babies…”

“…FGD 6: Participant 5 and FGD 1: Participants 2 and 5 had this to say,” “…this should be taught even at school to young women so that they grow up having this information of PCC and thus will know what to do before they fall pregnant…”

It is believed they will use the PCC services when informed. This quote highlights the perception that timely education is key to meaningful utilization. Analytical synthesis suggests that participants recognize a knowledge‐to‐action gap: Awareness must precede reproductive decision‐making to enhance proactive health behaviors. Collectively, these statements indicate that early dissemination of PCC information is perceived as foundational for improving maternal and neonatal outcomes.

#### 3.2.2. Theme 2: Potential Challenges Related to PCC Uptake and Utilization

The second theme was potential challenges related to PCC utilization. Four subthemes emerged under Theme 2, namely: lack of awareness about PCC by most WCA, fear of knowing about their HIV status, cultural issues regarding pregnancy that may have an impact on PCC utilization, and lack of support from a spouse.

##### 3.2.2.1. Subtheme 2.1: Lack of Awareness About PCC by Most Women of Reproductive Age

Several participants noted that PCC is largely unknown within the community:

“…This was evident through the following verbal quotes from WCA:

FGD 5: Participants 2 and 6 and FGD 2: Participants 4 and 5 shared the same sentiments and said that,”

“…The problem is that most women do not know about this [PCC], so even when coming to the clinic, they do not know that they can ask questions relating to their future pregnancies…”

FGD 3: Participant 2 and FGD 4: Participants 3 and 8 said, “…Since it is the first time we hear about it, I believe there are many more women who do not know about PCC and, therefore, will not know that they should come to the clinic before they fall pregnant…”

The interpretation here is that information gaps act as a primary barrier to utilization. Awareness is not just an individual‐level issue but reflects systemic gaps in health promotion and community outreach. The synthesis of multiple quotes across focus groups indicates a pattern of invisibility, where women may only access PCC reactively if health issues arise, rather than proactively seeking preventive care.

##### 3.2.2.2. Subtheme 2.2: Fear of Knowing About HIV Status

Being assessed and knowing about one′s HIV status instilled fear in some and was viewed as a challenge regarding the utilization of PCC. The following quotes are confirmation to that:

FGD 8: Participants 5 and 7 and Participants 3 and 8 of FGD 7 said:

“…Eh (pause) the other factor that can affect utilization is fear of being assessed for HIV status, [eish], testing for the first time is scary you know…”

FGD 1: Participants 1 and 4 echoed by FGD 5: Participants 3 and 5: “…Umm… other people will still not come to the clinic for fear of knowing their HIV status if it is also going to be tested during PCC…”

An analytical interpretation suggests that structural stigma and the fear of social consequences are embedded barriers. Participants′ narratives reveal that psychological and societal pressures intersect with health service utilization, demonstrating that PCC uptake is influenced not only by service availability but also by social and emotional factors.

##### 3.2.2.3. Subtheme 2.3: Existence of Cultural Issues Regarding Pregnancy That May Have an Impact on PCC Uptake and Utilization

Cultural norms governing disclosure of pregnancy intentions emerged as another barrier:

The following quotes are evidence of that.

“…FGD 7: Participants 2 and 4 and FGD 3: Participants 6 and 8 said”:

“…As Africans, we have a way of dealing with pregnancy and childbirth…”

“…While FGD 1: Participants 6 and 7 and FGD 7: Participants 1 and 7 had this to say”: “…You know our culture does not permit us to openly talk about our intentions to fall pregnant or to expose our pregnancy early…”

Synthesis indicates that cultural values shape health‐seeking behavior, potentially delaying or preventing preconception engagement. This finding highlights the importance of culturally sensitive PCC strategies and suggests that intervention design must respect local norms while promoting reproductive health.

##### 3.2.2.4. Subtheme 2.4: Lack of Spouse Support

The study′s findings demonstrated the value of spouse support regarding reproductive concerns. Participants highlighted that partner support is critical, both financially and emotionally, as demonstrated by the following quotes from participants:

“… FGD 4: Participants 2, 4, and 6 and FGD 6: Participants 2, 4, and 6 said,” “…Some women are not working, and they rely on their partners for transport money to get to the clinic, so if not given the money, they may not be able to honor their PCC appointments…”

“…FGD 2: Participants 6 and 7 and FGD 5: Participant 8 said”: “…I believe pregnancy and issues surrounding it are for the two people in the relationship, so if the woman does not get support and encouragement from her partner, she may feel discouraged to consult for PCC…”

“…FGD 8: Participants 2, 3, and 5 said in unison”: “…yeah, our partners or spouses also need to be part of everything that has to do with procreation, and it will help in making pivotal decisions regarding having children in terms of numbers and when to have them.”

Analysis shows that interpersonal dynamics directly influence utilization. PCC uptake is embedded within relational and economic contexts, emphasizing that male engagement and support structures are central to effective preconception interventions.

## 4. Discussion

The study findings revealed that women of reproductive age in Limpopo perceive PCC as a valuable preventive service. However, awareness gaps, fear of HIV testing, cultural norms, and lack of spouse support were identified as barriers to service utilization. Participants in the study reported that it would help women get pregnant while remaining healthy. This is a fundamental aspect of PCC, as it emphasizes identifying and addressing risk factors during the preconception period. The results of this study agree with those of Ukoha and Mtshali [13], in which participants thought PCC was useful and could reduce the risk of congenital defects and maternal and newborn deaths. Such sentiments were also echoed by Ndou et al. [17], who affirmed PCC as a strategic intervention to improve neonatal and delivery outcomes by optimizing maternal and fetal health before pregnancy and changing modifiable risk factors.

Participants in this study also highlighted the importance of educating young women before they start families. Adolescent pregnancy is a global concern with serious health consequences. The birth rate for girls aged 10–14 in 2022 globally was 1.5 per 1000 women [25]. On the other hand, in South Africa, between March 2021 and April 2022, 90,037 girls aged 10–19 gave birth [7]. Preventing adolescent pregnancy and childbirth is part of the SDGs 3, 4, 5, which advocate good health, gender equality, and quality education. To advance this goal and ensure that teenagers who do become pregnant do so in a healthy manner, PCCs play a crucial role. A study by Khekade et al. [26] indicates the importance of initiating increased awareness about sexual and reproductive health issues from the adolescent phase. The WHO [27] claimed that teenagers using PCC could significantly help to reduce the rate of maternal and newborn deaths. Therefore, the PCC information needs to be disseminated even to young girls at schools.

Although participants in this study perceived PCC as valuable and beneficial to improving maternal health, they also highlighted potential challenges to using PCC by women of reproductive age. The participants in the study mentioned a lack of awareness as a serious challenge to the utilization of PCC. Being aware of PCC and its benefits would help WCA make informed decisions about service utilization. Similar to findings by Carg et al. [28] and Woldeyohannes et al. [29], the current study highlights that a lack of awareness is a major barrier to PCC uptake. This suggests that improving knowledge is a critical step toward increasing utilization, emphasizing the need for targeted health education campaigns. Improving perinatal health outcomes is hindered by limited knowledge of PCC. The researchers believe raising awareness of PCC will probably encourage women of reproductive age to use it, enhancing perinatal outcomes. Supporting the findings, Carg et al. [28] and Sainafat et al. [30] also found that most women of reproductive age lack awareness about PCC, which negatively impacts their use of the PCC service. A report by Woldeyohannes et al. [29] also found that a lack of knowledge about PCC is a barrier to its use. Therefore, educating women of reproductive age and the public about PCC is critical to boosting the uptake of PCC services in the province of Limpopo′s chosen regions.

About 8.45 million South Africans live with HIV, which is an estimated 13.9% of the population [7]. One of the emphases of PCC is the treatment of HIV and other infections during the preconception period. Testing is the first step in accessing antiretrovirus treatment. The results of this study exposed fear of knowing about HIV status as a potential challenge to accessing PCC. Fear of HIV testing echoes observations by Okemo et al. [31] and Mwisongo et al. [32], where HIV‐related stigma and concerns over confidentiality limited engagement with reproductive health services. These findings underscore the need for confidential, supportive, and stigma‐sensitive approaches to PCC delivery. According to other reports by Okemo et al. [31] and M′hamdi et al. [33], obtaining one′s HIV test results, the stigma attached to it, and the discomfort of disclosing private information to medical professionals are obstacles to the uptake of HIV testing. The findings of the study by Mwisongo et al. [32] concluded that acceptability to HIV testing depended on an individual′s belief that their test results would be confidential. Thus, it is important to reassure clients about the confidentiality of the HIV testing and results [34].

The influence of cultural norms and lack of partner support corresponds with studies highlighting the role of interpersonal and community factors in reproductive health decisions [26, 35]. This indicates that PCC interventions must be culturally sensitive and actively involve male partners to enhance service uptake. Participants in this study viewed cultural issues surrounding pregnancy and childbirth as a challenge. Culture has a significant role in sexual reproductive health. It molds teenage girls′ sexual and reproductive health to be impacted by their sexual knowledge, attitudes, and practices [36]. The authors also confirm that cultural factors have an impact on how young females seek out sexual reproductive health services, such as contraception, maternity and childcare services, and access to and use of health information [37]. Hence, PCC should be provided in a culturally sensitive milieu for it to be acceptable [36]. It might be necessary for the older generation, known as the guardians of culture, to get involved in encouraging people who are fertile to seek prenatal and early pregnancy medical treatment.

The study participants said that spouse support was necessary for PCC uptake. Consequently, it was believed that the lack of partner support limited the application of PCC. In the study by Ryan et al. [34], they argued that men, when given reproductive health information, can have a pivotal role in supporting their partners in reproductive health issues, including contraception decisions [36]. This was echoed by Achen et al. [38], who proposed that more effort should be directed toward involving men in PCC. On the other hand, a report by Khekade et al. [26] advocate that women should be encouraged to make decisions regarding reproductive health matters with the support of other family members. Although previous studies have examined perceptions of PCC, the current research provides context‐specific insights from Limpopo province, particularly regarding how cultural norms and male partner involvement intersect with service utilization. These insights can inform locally relevant strategies to improve PCC implementation.

## 5. Conclusion

This study demonstrates that women of reproductive age in the selected districts of Limpopo perceive PCC as a valuable service that can promote maternal and newborn health. Key barriers to PCC uptake were identified, including limited awareness, fear of HIV testing, cultural norms surrounding pregnancy, and lack of partner support. These findings underscore the importance of addressing both sociocultural and structural factors to enhance service utilization.

## 6. Recommendations

Based on the study findings, several recommendations are proposed to improve the uptake and use of PCC services in Limpopo province.

• Increase awareness and education: implement community campaigns, school programs, and outreach initiatives to educate women of reproductive age about PCC, using culturally appropriate information in local languages such as Tshivenda, Xitsonga, and Sepedi.
• Promote partner and family involvement: encourage male partners to participate in reproductive health education and support joint decision‐making about pregnancy planning and reproductive health.
• Use digital health tools: introduce mobile apps, SMS platforms, and online resources to provide accessible PCC information, ensuring they are user‐friendly and integrated with primary healthcare services.
• Strengthen health system integration: integrate PCC into routine primary healthcare and maternal services, and train healthcare providers to deliver PCC effectively with culturally sensitive communication.
• Policy and advocacy: support policies that recognize PCC as a key component of reproductive health services and allocate resources to expand access, especially in rural areas.

## 7. Limitations

The study was conducted only in three districts (Capricorn, Mopani, and Vhembe) in Limpopo province. Findings may not be generalizable to other provinces in South Africa or to any district where sociocultural and structural dynamics differ. Participants′ perceptions reflect the period during which data were collected. Changes in awareness, healthcare policy, or service availability over time may influence PCC perceptions differently in the future.

**Nomenclature**
CHCs: Community health centers
FGDs: Focus group discussions
HIV: Human immunodeficiency virus
PCC: Preconception care
SA: South Africa
UNICEF: United Nations International Children′s Emergency Fund
WHO: World Health Organization

## Funding

This study was supported by South African Medical Research Council (10.13039/501100001322; 57009).

## Disclosure

An earlier version of this work was presented by the principal investigator Prof. Malwela T., as part of a progress report in the regional grant (RCDI) holders conference held at Sefako Makgatho Health Sciences University on October 20–21, 2025. Research Capacity Development, Regional Grant Holders, Conference 2025, October 20–21, 2025.

## Conflicts of Interest

The authors declare no conflicts of interest.

## Data Availability

The data that support the findings of this study are openly available in UNIVEN IR at https://univendspace.univen.ac.za/home.
